# (2*SR*,3*SR*)-Isopropyl 3-{[dimeth­yl(phenyl)­sil­yl]meth­yl}-2-hy­droxy-2-vinyl­pent-4-enoate

**DOI:** 10.1107/S1600536810044818

**Published:** 2010-11-06

**Authors:** Bjoern Nelson, Markus Schürmann, Hans Preut, Martin Hiersemann

**Affiliations:** a Fakultät Chemie, Technische Universität Dortmund, Otto-Hahn-Strasse 6, 44221 Dortmund, Germany

## Abstract

The relative configuration of the title compound, C_19_H_28_O_3_Si, which was synthesized using a dienolate-[2,3]-Wittig rearrangement, was corroborated by single-crystal X-ray diffraction analysis. The Si—C bond distances are in the range 1.858 (2)–1.880 (2) Å and an intra­molecular O—H⋯O hydrogen bond helps to stabilize the mol­ecular conformation.

## Related literature

For background literature on Wittig rearrangements, see: Abraham *et al.* (2003 ▶); Hiersemann (1999 ▶, 2000 ▶); Lauterbach *et al.* (1999 ▶); Le Menez *et al.* (1995 ▶).
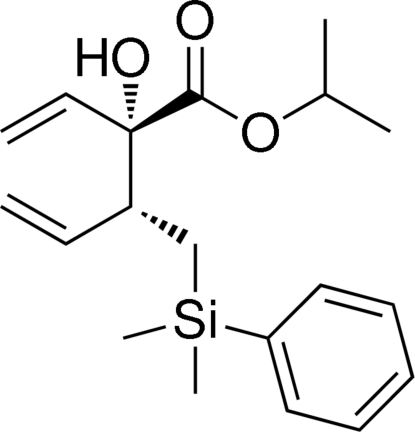


## Experimental

### 

#### Crystal data

C_19_H_28_O_3_Si
*M* *_r_* = 332.50
Monoclinic, 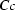

*a* = 18.4311 (15) Å
*b* = 12.0676 (10) Å
*c* = 8.8508 (6) Å
β = 95.366 (7)°
*V* = 1960.0 (3) Å^3^
*Z* = 4
Mo *K*α radiation
μ = 0.13 mm^−1^
*T* = 173 K
0.44 × 0.12 × 0.10 mm

#### Data collection

Oxford Xcalibur S CCD diffractometer
Absorption correction: multi-scan (*CrysAlis RED*; Oxford Diffraction, 2008 ▶) *T*_min_ = 0.92, *T*_max_ = 1.00
6497 measured reflections
3425 independent reflections
2466 reflections with *I* > 2σ(*I*)
*R*_int_ = 0.035

#### Refinement

*R*[*F* ^2^ > 2σ(*F* ^2^)] = 0.040
*wR*(*F* ^2^) = 0.044
*S* = 1.01
3425 reflections
213 parameters
2 restraints
H-atom parameters constrained
Δρ_max_ = 0.28 e Å^−3^
Δρ_min_ = −0.21 e Å^−3^
Absolute structure: Flack (1983 ▶), 1066 Friedel pairs
Flack parameter: 0.09 (9)

### 

Data collection: *CrysAlis CCD* (Oxford Diffraction, 2008 ▶); cell refinement: *CrysAlis CCD*; data reduction: *CrysAlis CCD*; program(s) used to solve structure: *SHELXS97* (Sheldrick, 2008 ▶); program(s) used to refine structure: *SHELXL97* (Sheldrick, 2008 ▶); molecular graphics: *SHELXTL-Plus* (Sheldrick, 2008 ▶); software used to prepare material for publication: *SHELXL97* and *PLATON* (Spek, 2009 ▶).

## Supplementary Material

Crystal structure: contains datablocks I, global. DOI: 10.1107/S1600536810044818/hb5668sup1.cif

Structure factors: contains datablocks I. DOI: 10.1107/S1600536810044818/hb5668Isup2.hkl

Additional supplementary materials:  crystallographic information; 3D view; checkCIF report

## Figures and Tables

**Table 1:** Hydrogen-bond geometry (Å, °)

*D*—H⋯*A*	*D*—H	H⋯*A*	*D*⋯*A*	*D*—H⋯*A*
O3—H3⋯O2	0.84	2.17	2.664 (2)	118

**Fig. 1.**
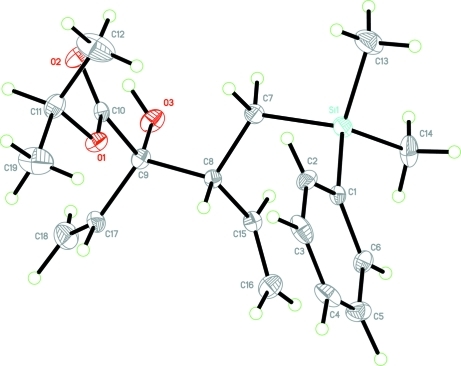
The molecular structure of the title compound, with displacement ellipsoids shown at the 30% probability level.

## supplementary crystallographic information

### Comment

The title compound I was synthesized from allylic alcohol (Le Menez *et al.*, 1995), followed by diastereoselective dienolate-[2,3]-Wittig rearrangement (Lauterbach *et al.*, 1999). Fig. 1 depicts the structure of isolated racemic major diastereomer of I. The relative configuration of the stereogenic centers in I can be attributed to the stereochemical course of the dienolate-[2,3]-Wittig rearrangement (Hiersemann, 1999, 2000; Abraham *et al.*, 2003).

### Experimental

To a cooled (195 K) solution of LDA [prepared *in situ* from NEt_3_ (4.2 mmol, 0.6 ml, 1.4 eq) and *n*-BuLi (2.3 *M* in hexanes, 4.2 mmol, 1.8 ml, 1.4 eq) in THF (15 ml, 3.5 ml/mmol NEt_3_) was added dropwise a pre-cooled (195 K) solution of the allyl vinyl ether II ((*Z*,*E*)/(*E*,*E*) = 3/2, 3.0 mmol, 1 g, 1 eq) in THF (6 ml, 2 ml/mmol II). The reaction mixture was stirred at 195 K for 30 min, warmed to 273 K and stirred for an additional 60 min. After the addition of saturated aqueous NH_4_Cl solution the aqueous layer was extracted with CH_2_Cl_2_ (3×) and the combined organic phases were dried (MgSO_4_) and concentrated under reduced pressure. Purification by flash chromatography (cyclohexane/ethyl acetate 100/1 to 50/1) afforded the 1,5-hexadiene I (813 mg, 2.44 mmol, 82%) as a mixture of diastereomers (dr = 86/14) as colourless crystals. Subsequent recrystallization of I by vapour diffusion technique from isohexane and ethyl acetate provided colourless needles of the major diastereomer of (I) suitable for an X-ray crystal structure analysis.

*R_f_* 0.60 (cyclohexane/ethyl acetate 5/1); ^1^H NMR (CDCl_3_, 400 MHZ, δ): 0.23 (s, 3H^minor^, CH_3_), 0.24 (s, 3H^major^, CH_3_), 0.26 (s, 3H^minor^, CH_3_), 0.27 (s, 3H^major^, CH_3_), 0.58 (dd, *J* = 14.6, 2.0 Hz, 2H^major^, CH_2_), 0.86 (dd, *J* = 15.1, 12.1 Hz, 2H^minor, CH^_2_), 1.03 (dd, *J* = 14.8, 2.3 Hz, 2H^minor^, CH_2_), 1.10 (dd, *J* = 14.6, 12.6 Hz, 2H^major^, CH_2_), 1.20–1.24 (m, 6H, 2 × CH_3_), 2.50 (ddd, *J* = 12.1, 9.8, 2.3 Hz, 1H^minor^, CH), 2.59 (ddd, *J* = 12.6, 9.8, 2.0 Hz, 1H^major^, CH), 3.29 (s, 1H^minor^, OH), 3.33 (s, 1H^major^, OH), 4.88–5.07 (m, 3 × 1H, CH), 5.13 (dd, *J* = 10.5, 1,5 Hz, 1H^major^, H_2_C═), 5.23 (dd, *J* = 10.5, 1,5 Hz, 1H^minor^, H_2_C═), 5.37 (dd, *J* = 17.1, 1,5 Hz, 1H^major^, H_2_C═), 5.42–5.52 (m, 1H^major^+1H^minor^, CH), 5.56–5.65 (m, 1H^minor^, CH), 5.78 (dd, *J* = 16.8, 10,3 Hz, 1H^minor^, H_2_C═), 5.81 (dd, *J* = 17.1, 10.5 Hz, 1H^major^, H_2_C═), 7.31–7.35 (m, 3H, 3 × CH^Ar^), 7.42–7.48 (m, 2H, 2 × CH^Ar^); ^13^C NMR (CDCl_3_, 101 MHz, δ): -2.4 (CH_3_^minor^), -2.3 (CH_3_^major^), -1.5 (CH_3_^minor^), -1.4 (CH_3_^major^), 13.8 (CH_2_^minor^), 15.8 (CH_2_^major), 21.8 (CH^_3_), 21.8 (CH_3_^minor^), 21.9 (CH_2_^major), ^47.6 (CH^minor^), 47.8 (CH^major^), 70.3 (CH), 80.9 (C^major^), 81.0 (C^minor^), 115.3 (CH_2_^major^), 116.6 (CH_2_^minor^), 117.4 (CH_2_^minor^), 117.8 (CH_2_^major^), 127.7 (CH^minor^), 127.8 (CH^major^), 128.9 (CH^minor^), 129.0 (CH^major^), 133.7 (CH^major^), 133.8 (CH^minor^), 138.0 (CH^minor^), 138.0 (CH^major^), 138.3 (CH^major^), 138.8 (CH^minor^), 139.4 (C), 174.6 (C); IR (cm^-1^): 3505 (br,s) (ν OH), 3070 (w), 3050 (w), 3020 (w), 2980 (*m*), 2955 (*m*), 2920 (w), 1725 (*s*) (ν C═O), 1640 (w), 1620 (w), 1470 (w), 1455 (w), 1430 (*m*), 1400 (w), 1390 (w), 1375 (*m*), 1260 (*s*), 1190 (*s*), 1140 (*m*), 1105 (*s*), 1085 (*m*); Anal. Calcd. for C_19_H_28_O_3_Si: C, 68.6; H, 8.5; Found: C, 68.6; H,8.2; *M* = 332.51 g/mol.

### Crystal data

**Table:**

C_19_H_28_O_3_Si	*F*(000) = 720
*M**_r_* = 332.50	*D*_x_ = 1.127 Mg m^−^^3^
Monoclinic, *C**c*	Mo *K*α radiation, λ = 0.71073 Å
Hall symbol: C -2yc	Cell parameters from 2622 reflections
*a* = 18.4311 (15) Å	θ = 2.2–29.1°
*b* = 12.0676 (10) Å	µ = 0.13 mm^−^^1^
*c* = 8.8508 (6) Å	*T* = 173 K
β = 95.366 (7)°	Block, colourless
*V* = 1960.0 (3) Å^3^	0.44 × 0.12 × 0.10 mm
*Z* = 4	

### Data collection

**Table:**

Oxford Xcalibur S CCD diffractometer	3425 independent reflections
Radiation source: Enhance (Mo) X-ray Source	2466 reflections with *I* > 2σ(*I*)
graphite	*R*_int_ = 0.035
Detector resolution: 16.0560 pixels mm^-1^	θ_max_ = 25.5°, θ_min_ = 2.2°
ω scans	*h* = −21→22
Absorption correction: multi-scan (*CrysAlis RED*; Oxford Diffraction, 2008)	*k* = −14→14
*T*_min_ = 0.92, *T*_max_ = 1.00	*l* = −10→10
6497 measured reflections	

### Refinement

**Table:**

Refinement on *F*^2^	Secondary atom site location: difference Fourier map
Least-squares matrix: full	Hydrogen site location: inferred from neighbouring sites
*R*[*F*^2^ > 2σ(*F*^2^)] = 0.040	H-atom parameters constrained
*wR*(*F*^2^) = 0.044	*w* = 1/[σ^2^(*F*_o_^2^)
*S* = 1.00	(Δ/σ)_max_ = 0.001
3425 reflections	Δρ_max_ = 0.28 e Å^−^^3^
213 parameters	Δρ_min_ = −0.21 e Å^−^^3^
2 restraints	Absolute structure: Flack (1983), with how many Friedel pairs?
Primary atom site location: structure-invariant direct methods	Flack parameter: 0.09 (9)

### Special details

**Table:**

Geometry. All e.s.d.'s (except the e.s.d. in the dihedral angle between two l.s. planes) are estimated using the full covariance matrix. The cell e.s.d.'s are taken into account individually in the estimation of e.s.d.'s in distances, angles and torsion angles; correlations between e.s.d.'s in cell parameters are only used when they are defined by crystal symmetry. An approximate (isotropic) treatment of cell e.s.d.'s is used for estimating e.s.d.'s involving l.s. planes.
Refinement. Refinement of *F*^2^ against ALL reflections. The weighted *R*-factor *wR* and goodness of fit *S* are based on *F*^2^, conventional *R*-factors *R* are based on *F*, with *F* set to zero for negative *F*^2^. The threshold expression of *F*^2^ > σ(*F*^2^) is used only for calculating *R*-factors(gt) *etc*. and is not relevant to the choice of reflections for refinement. *R*-factors based on *F*^2^ are statistically about twice as large as those based on *F*, and *R*- factors based on ALL data will be even larger.

### Fractional atomic coordinates and isotropic or equivalent isotropic displacement parameters (Å^2^)

**Table:**

	*x*	*y*	*z*	*U*_iso_*/*U*_eq_	
Si	0.22320 (4)	0.22241 (6)	0.31540 (6)	0.02606 (19)	
C1	0.17453 (12)	0.2779 (2)	0.4769 (2)	0.0203 (6)	
O1	0.42490 (8)	0.24617 (13)	0.68996 (16)	0.0266 (5)	
O2	0.50238 (10)	0.17136 (15)	0.53585 (17)	0.0373 (5)	
C2	0.17399 (13)	0.2176 (2)	0.6094 (2)	0.0271 (7)	
H2	0.1974	0.1474	0.6173	0.033*	
O3	0.46978 (10)	0.32269 (15)	0.32107 (15)	0.0304 (5)	
H3	0.4988	0.2689	0.3297	0.046*	
C3	0.13979 (16)	0.2578 (2)	0.7307 (3)	0.0375 (8)	
H3A	0.1404	0.2155	0.8213	0.045*	
C4	0.10492 (15)	0.3585 (2)	0.7211 (3)	0.0343 (8)	
H4	0.0810	0.3856	0.8042	0.041*	
C5	0.10492 (16)	0.4195 (2)	0.5904 (3)	0.0353 (7)	
H5	0.0811	0.4894	0.5828	0.042*	
C6	0.13942 (14)	0.3794 (2)	0.4699 (2)	0.0263 (7)	
H6	0.1391	0.4225	0.3800	0.032*	
C7	0.32344 (12)	0.2331 (2)	0.3641 (2)	0.0251 (6)	
H7A	0.3377	0.1759	0.4413	0.030*	
H7B	0.3475	0.2140	0.2722	0.030*	
C8	0.35493 (12)	0.3452 (2)	0.4244 (2)	0.0200 (6)	
H8	0.3331	0.3620	0.5211	0.024*	
C9	0.43837 (13)	0.3388 (2)	0.4608 (2)	0.0224 (6)	
C10	0.45930 (13)	0.2409 (2)	0.5653 (3)	0.0213 (7)	
C11	0.44411 (16)	0.1640 (2)	0.8072 (3)	0.0338 (8)	
H11	0.4981	0.1532	0.8184	0.041*	
C12	0.40720 (19)	0.0556 (2)	0.7641 (3)	0.0637 (11)	
H12A	0.4224	0.0303	0.6667	0.096*	
H12B	0.3542	0.0659	0.7553	0.096*	
H12C	0.4210	0.0001	0.8425	0.096*	
C13	0.20207 (15)	0.0718 (2)	0.2902 (3)	0.0478 (9)	
H13A	0.2285	0.0419	0.2082	0.072*	
H13B	0.1496	0.0621	0.2645	0.072*	
H13C	0.2170	0.0322	0.3847	0.072*	
C14	0.19202 (14)	0.2949 (2)	0.1357 (2)	0.0411 (8)	
H14A	0.1999	0.3748	0.1485	0.062*	
H14B	0.1400	0.2805	0.1098	0.062*	
H14C	0.2197	0.2677	0.0540	0.062*	
C15	0.33501 (13)	0.4375 (2)	0.3156 (2)	0.0225 (7)	
H15	0.3503	0.4310	0.2164	0.027*	
C16	0.29856 (14)	0.5262 (2)	0.3452 (3)	0.0367 (8)	
H16A	0.2823	0.5361	0.4430	0.044*	
H16B	0.2883	0.5809	0.2690	0.044*	
C17	0.46867 (14)	0.4419 (2)	0.5398 (3)	0.0278 (7)	
H17	0.4481	0.4647	0.6292	0.033*	
C18	0.52116 (15)	0.5018 (2)	0.4934 (3)	0.0445 (8)	
H18A	0.5429	0.4813	0.4044	0.053*	
H18B	0.5376	0.5659	0.5488	0.053*	
C19	0.42038 (18)	0.2118 (2)	0.9503 (3)	0.0549 (10)	
H19A	0.4444	0.2834	0.9707	0.082*	
H19B	0.4338	0.1610	1.0348	0.082*	
H19C	0.3674	0.2221	0.9394	0.082*	

### Atomic displacement parameters (Å^2^)

**Table:**

	*U*^11^	*U*^22^	*U*^33^	*U*^12^	*U*^13^	*U*^23^
Si	0.0214 (5)	0.0303 (5)	0.0268 (4)	−0.0022 (5)	0.0040 (3)	−0.0070 (4)
C1	0.0163 (16)	0.0245 (16)	0.0198 (14)	−0.0033 (15)	−0.0006 (12)	0.0011 (14)
O1	0.0297 (12)	0.0260 (12)	0.0247 (9)	0.0058 (9)	0.0058 (8)	0.0037 (8)
O2	0.0312 (14)	0.0389 (14)	0.0432 (12)	0.0143 (11)	0.0103 (10)	0.0044 (10)
C2	0.0278 (18)	0.0244 (16)	0.0290 (14)	0.0001 (15)	0.0017 (13)	0.0041 (14)
O3	0.0249 (13)	0.0384 (14)	0.0300 (10)	0.0074 (10)	0.0143 (9)	0.0014 (9)
C3	0.047 (2)	0.044 (2)	0.0217 (14)	−0.0211 (17)	0.0032 (13)	0.0031 (14)
C4	0.033 (2)	0.039 (2)	0.0348 (17)	−0.0160 (16)	0.0202 (14)	−0.0153 (15)
C5	0.0300 (19)	0.0297 (19)	0.0481 (16)	0.0024 (15)	0.0126 (14)	−0.0056 (16)
C6	0.0240 (18)	0.0287 (18)	0.0261 (14)	0.0005 (14)	0.0029 (13)	0.0064 (13)
C7	0.0236 (16)	0.0261 (17)	0.0256 (13)	−0.0003 (14)	0.0025 (11)	−0.0014 (13)
C8	0.0201 (18)	0.0242 (17)	0.0164 (11)	0.0007 (13)	0.0050 (11)	−0.0020 (12)
C9	0.0176 (18)	0.0270 (18)	0.0234 (14)	0.0007 (13)	0.0065 (12)	0.0019 (13)
C10	0.0158 (19)	0.0235 (19)	0.0239 (14)	−0.0067 (14)	−0.0018 (13)	−0.0014 (13)
C11	0.032 (2)	0.038 (2)	0.0309 (16)	0.0057 (16)	−0.0018 (14)	0.0100 (15)
C12	0.104 (3)	0.035 (2)	0.0538 (19)	−0.021 (2)	0.014 (2)	0.0047 (17)
C13	0.039 (2)	0.044 (2)	0.0622 (19)	−0.0111 (16)	0.0144 (16)	−0.0227 (17)
C14	0.0227 (18)	0.073 (2)	0.0273 (14)	−0.0045 (17)	−0.0008 (13)	−0.0037 (15)
C15	0.0206 (17)	0.0261 (18)	0.0211 (13)	−0.0022 (14)	0.0044 (12)	0.0028 (13)
C16	0.039 (2)	0.034 (2)	0.0363 (16)	0.0047 (16)	0.0031 (15)	0.0064 (14)
C17	0.0202 (18)	0.029 (2)	0.0336 (15)	−0.0023 (14)	0.0000 (13)	−0.0064 (14)
C18	0.040 (2)	0.037 (2)	0.0553 (19)	−0.0103 (18)	−0.0019 (16)	−0.0032 (16)
C19	0.092 (3)	0.046 (2)	0.0287 (16)	0.004 (2)	0.0137 (17)	0.0057 (16)

### Geometric parameters (Å, °)

**Table:**

Si—C14	1.859 (2)	C9—C17	1.508 (3)
Si—C7	1.862 (2)	C9—C10	1.527 (3)
Si—C13	1.868 (2)	C11—C19	1.494 (3)
Si—C1	1.880 (2)	C11—C12	1.507 (3)
C1—C2	1.381 (3)	C11—H11	1.0000
C1—C6	1.385 (3)	C12—H12A	0.9800
O1—C10	1.325 (2)	C12—H12B	0.9800
O1—C11	1.454 (3)	C12—H12C	0.9800
O2—C10	1.200 (3)	C13—H13A	0.9800
C2—C3	1.383 (3)	C13—H13B	0.9800
C2—H2	0.9500	C13—H13C	0.9800
O3—C9	1.427 (2)	C14—H14A	0.9800
O3—H3	0.8400	C14—H14B	0.9800
C3—C4	1.373 (3)	C14—H14C	0.9800
C3—H3A	0.9500	C15—C16	1.303 (3)
C4—C5	1.371 (3)	C15—H15	0.9500
C4—H4	0.9500	C16—H16A	0.9500
C5—C6	1.379 (3)	C16—H16B	0.9500
C5—H5	0.9500	C17—C18	1.304 (3)
C6—H6	0.9500	C17—H17	0.9500
C7—C8	1.547 (3)	C18—H18A	0.9500
C7—H7A	0.9900	C18—H18B	0.9500
C7—H7B	0.9900	C19—H19A	0.9800
C8—C15	1.496 (3)	C19—H19B	0.9800
C8—C9	1.543 (3)	C19—H19C	0.9800
C8—H8	1.0000		
			
C14—Si—C7	112.71 (11)	O2—C10—C9	123.0 (2)
C14—Si—C13	108.16 (12)	O1—C10—C9	110.7 (2)
C7—Si—C13	106.64 (12)	O1—C11—C19	105.7 (2)
C14—Si—C1	110.59 (11)	O1—C11—C12	109.7 (2)
C7—Si—C1	109.45 (10)	C19—C11—C12	112.8 (2)
C13—Si—C1	109.15 (12)	O1—C11—H11	109.5
C2—C1—C6	117.6 (2)	C19—C11—H11	109.5
C2—C1—Si	119.99 (19)	C12—C11—H11	109.5
C6—C1—Si	122.36 (17)	C11—C12—H12A	109.5
C10—O1—C11	117.3 (2)	C11—C12—H12B	109.5
C3—C2—C1	121.0 (2)	H12A—C12—H12B	109.5
C3—C2—H2	119.5	C11—C12—H12C	109.5
C1—C2—H2	119.5	H12A—C12—H12C	109.5
C9—O3—H3	109.5	H12B—C12—H12C	109.5
C4—C3—C2	120.5 (2)	Si—C13—H13A	109.5
C4—C3—H3A	119.8	Si—C13—H13B	109.5
C2—C3—H3A	119.8	H13A—C13—H13B	109.5
C5—C4—C3	119.3 (2)	Si—C13—H13C	109.5
C5—C4—H4	120.3	H13A—C13—H13C	109.5
C3—C4—H4	120.3	H13B—C13—H13C	109.5
C4—C5—C6	120.1 (2)	Si—C14—H14A	109.5
C4—C5—H5	120.0	Si—C14—H14B	109.5
C6—C5—H5	120.0	H14A—C14—H14B	109.5
C1—C6—C5	121.5 (2)	Si—C14—H14C	109.5
C1—C6—H6	119.2	H14A—C14—H14C	109.5
C5—C6—H6	119.2	H14B—C14—H14C	109.5
C8—C7—Si	118.22 (17)	C16—C15—C8	125.6 (2)
C8—C7—H7A	107.8	C16—C15—H15	117.2
Si—C7—H7A	107.8	C8—C15—H15	117.2
C8—C7—H7B	107.8	C15—C16—H16A	120.0
Si—C7—H7B	107.8	C15—C16—H16B	120.0
H7A—C7—H7B	107.1	H16A—C16—H16B	120.0
C15—C8—C9	110.6 (2)	C18—C17—C9	124.4 (2)
C15—C8—C7	111.55 (18)	C18—C17—H17	117.8
C9—C8—C7	111.3 (2)	C9—C17—H17	117.8
C15—C8—H8	107.8	C17—C18—H18A	120.0
C9—C8—H8	107.8	C17—C18—H18B	120.0
C7—C8—H8	107.8	H18A—C18—H18B	120.0
O3—C9—C17	110.6 (2)	C11—C19—H19A	109.5
O3—C9—C10	108.6 (2)	C11—C19—H19B	109.5
C17—C9—C10	107.24 (19)	H19A—C19—H19B	109.5
O3—C9—C8	107.62 (18)	C11—C19—H19C	109.5
C17—C9—C8	112.0 (2)	H19A—C19—H19C	109.5
C10—C9—C8	110.8 (2)	H19B—C19—H19C	109.5
O2—C10—O1	126.3 (3)		
			
C14—Si—C1—C2	162.39 (19)	C7—C8—C9—O3	66.2 (2)
C7—Si—C1—C2	−72.9 (2)	C15—C8—C9—C17	63.3 (2)
C13—Si—C1—C2	43.5 (2)	C7—C8—C9—C17	−172.12 (17)
C14—Si—C1—C6	−18.7 (2)	C15—C8—C9—C10	−177.0 (2)
C7—Si—C1—C6	106.0 (2)	C7—C8—C9—C10	−52.4 (2)
C13—Si—C1—C6	−137.6 (2)	C11—O1—C10—O2	4.0 (4)
C6—C1—C2—C3	−0.3 (3)	C11—O1—C10—C9	−174.6 (2)
Si—C1—C2—C3	178.7 (2)	O3—C9—C10—O2	8.1 (3)
C1—C2—C3—C4	0.7 (4)	C17—C9—C10—O2	−111.4 (3)
C2—C3—C4—C5	−0.7 (4)	C8—C9—C10—O2	126.1 (3)
C3—C4—C5—C6	0.3 (4)	O3—C9—C10—O1	−173.21 (19)
C2—C1—C6—C5	−0.1 (4)	C17—C9—C10—O1	67.3 (2)
Si—C1—C6—C5	−179.0 (2)	C8—C9—C10—O1	−55.2 (3)
C4—C5—C6—C1	0.1 (4)	C10—O1—C11—C19	159.6 (2)
C14—Si—C7—C8	74.11 (18)	C10—O1—C11—C12	−78.5 (3)
C13—Si—C7—C8	−167.35 (17)	C9—C8—C15—C16	−114.6 (3)
C1—Si—C7—C8	−49.40 (18)	C7—C8—C15—C16	121.0 (3)
Si—C7—C8—C15	−56.7 (2)	O3—C9—C17—C18	−6.0 (4)
Si—C7—C8—C9	179.26 (14)	C10—C9—C17—C18	112.3 (3)
C15—C8—C9—O3	−58.4 (3)	C8—C9—C17—C18	−126.0 (3)

### Hydrogen-bond geometry (Å, °)

**Table:**

*D*—H···*A*	*D*—H	H···*A*	*D*···*A*	*D*—H···*A*
O3—H3···O2	0.84	2.17	2.664 (2)	118
